# Clinical outcomes of single implant supported crowns versus 3-unit implant-supported fixed dental prostheses in Dubai Health Authority: a retrospective study

**DOI:** 10.1186/s12903-021-01530-2

**Published:** 2021-04-01

**Authors:** Sara Hussain Alhammadi, Girvan Burnside, Alexander Milosevic

**Affiliations:** 1 Hamdan Bin Mohamed College of Dental Medicine, Mohamed Bin Rashid University of Medicine and Health Sciences (MBRU), Dubai, UAE; 2 Department of Health Data Science, University of Liverpool, Waterhouse Building Block F, 1-5 Brownlow Street, Liverpool, L69 3GL UK

**Keywords:** Dental implants, Bone loss, Technical complications

## Abstract

**Background:**

This study assessed retrospectively the clinical outcomes of single implant-supported crowns and implant-supported fixed dental prostheses (FDPs).

**Methods:**

This case series compared biological and technical complications in single implant-supported crowns and implant-supported bridges in a time framed sample of all patients who received dental implants between 2009 and 2016 in Dubai Health Authority. Only 3-unit implant-supported prostheses (FDPs) with one intervening pontic and an implant each end were included for comparison to single crown supported implants. Cantilevered implants, implant-supported dentures and cases involving bone grafts or sinus lifts were excluded. The primary outcome measure was marginal bone loss, measured on digital radiographs taken after prosthesis placement at baseline and one year after implant loading, whilst peri-implantitis and technical complications were secondary outcomes. Mixed regression models adjusted for clustering of implants within patients was used for patient and implant factor associations.

**Results:**

A total of 454 patients (152 males; 302 females) had 1673 implants. The mean age of males (53.7 years, SD 14.6) was significantly greater than females (49.3 years, SD 12.9, p < 0.001). Mean mesial bone loss on the FDPs was significantly greater at 1 year (1.14 mm, SD 0.63) compared with the mesial surface of single implant-supported crowns (0.30 mm, SD 0.43, p < 0.001). Mean distal bone loss was also significantly greater at 1 year on the distal surfaces of implants supporting bridgework (1.29 mm, SD 0.71) compared with distal surfaces on single implant-supported crowns (0.36 mm, SD 0.54, p < 0.001). Mean marginal bone loss mesially and distally around implants placed in the lower anterior sextant was significantly greater compared to all other sites (p < 0.001). Bone loss by gender, patient’s age and medical condition was not different between the 2 implant groups. Screw loosening was the main technical complication (11.5%) whilst peri-implantitis occurred rarely (0.5%). The 66 cement retained implants had significantly more complications compared to the 1607 screw retained implants (p < 0.001).

**Conclusions:**

Mean marginal bone loss around the supporting implants of FDPs (3-unit fixed bridgework) was greater than on single implant-supported crowns at one year after implant loading. Position in the mouth was associated with bone loss. Biological and technical complications occurred rarely.

## Background

The survival and/or complications of single implant-supported crowns have been compared to implant-supported bridges (fixed dental prostheses) and reviewed systematically [1]. The survival of single implant-supported crowns over 5 and 10-years was reported to be 94.5% and 89.4% respectively, which was greater than implant-supported prostheses at 10-years (86.7%) [1]. Studies have compared biological and prosthetic complications of implant-supported crowns with tooth supported prostheses but few studies have compared single implant-supported crowns with the 3-unit fixed–fixed implant-supported bridge or FDP (fixed dental prosthesis). Pooled success rates at 5 years for implant-supported single crowns was higher at 95% compared to natural tooth supported FDPs at 84% (95% CI 79–89%) [2]. Furthermore, the estimated cumulative survival and complications of posterior 3-unit FDPs were not different at 15 years when compared with posterior implant-supported crowns but anterior implant-supported crowns survived significantly better [3]. A recent systematic review and meta-analysis concluded that implant-supported 3-unit FDPs had survival rates no different to those of tooth-supported 3-unit FDPs [4]. Only one study has directly compared 3 adjacent single implant-supported crowns (non-splinted), 3 adjacent splinted (connected) implant-supported crowns and 3-unit implant-supported FDPs with one intervening pontic [5]. The non-splinted and splinted crowns had worse survival than 3-unit FDPs, termed implant-supported bridges in the study [5]. Prosthodontic complications and peri-implantitis were the lowest in the FDP group with a significant three-fold increased risk of implant related complications in the 3 adjacent non-splinted single implant crowns compared to implant-supported bridges [5]. Splinted implants were reported to have improved load distribution and reduced marginal bone loss using finite element analysis and photo-elastic modelling [6, 7]. The rationale for splinting has been questioned in light of recent developments in dental implant design, implant surface properties and improved surgical techniques [8–10].

Plaque accumulation can lead to calculus formation, peri-mucositis or peri-implantitis with subsequent bone loss around the implants [11]. The primary advantage of a single implant-supported crown is ease of effective interproximal hygiene, better passivity of the framework and ease of removal or repair compared to splinted or connected units [9].

Forces transferred to implants are concentrated in the coronal 2 to 3 mm of crestal bone surrounding an implant [12]. Additionally, overloading may produce micro-fractures of crestal bone, mechanical failure and fracture of the implant or fatigue fractures of the prosthetic components [13]. The mandible has been shown to deform between 420 µm and 1.06 mm during function [14, 15]. Thus, restoration with single crown supported implants may minimize screw loosening or fractures as a result of mandibular flexion [16], whilst a fixed–fixed design may reduce stress concentration at the crestal bone margin resulting in better bone stability. Furthermore, fabrication of a passive fit of the connected prosthetic super-structure on implants is technically more difficult to achieve than is the case with individual implant-supported crowns [6].

The estimated survival of 2116 implant-supported fixed partial dentures after 6–7 years for the 10 studies in a meta-analysis was 93.6% and 97.5% for single implants [17]. A 10-year randomized controlled trial assessed marginal bone around multiple adjacent dental implants restored with connected and single prostheses found a mean 1.2 mm marginal bone loss around connected dental implants compared to a mean 1.3 mm marginal bone loss around single dental implants, but the 0.1 mm difference was not considered clinically significant [7]. Others have reported no significant difference in bone loss around single and connected dental implants or that connected implants showed greater crestal bone loss of 0.2 mm compared to single implants [4, 18].

A 10-Year retrospective study compared the prosthetic complications in single implant-supported crowns to fixed–fixed implant-supported bridges and found that fixed–fixed implant-supported bridges had more technical complications compared to single implants [19]. A quarter had prosthetic complications, of which chipped ceramic was the most frequent followed by screw loosening and de-cementation [19]. Others found that 38.7% of fixed–fixed implant-supported bridges had complications after 5 years [1].

The lack of literature regarding the clinical outcomes of implants placed in Dubai prompted this investigation which aimed to assess and compare the clinical outcomes of single implant-supported crowns to 3-unit fixed–fixed implant-supported bridges placed in Al Badaa Dental Centre in Dubai Health Authority (DHA) between January 2009 and December 2016. The primary outcome measure was crestal bone height and complications such as implantitis, mobility, screw loosening and de-cementation were secondary outcomes. This study asks the question, ‘Is bone loss in 3-unit implant-supported dental prostheses (FDPs) with one intervening pontic different to single implant-supported crowns one year after prosthesis placement/loading? A secondary aim was to determine’What factors influence clinical outcomes in implant-supported bridges (FDPs) and implant-supported single crowns?’.

## Methods

The study was a retrospective descriptive study of a time framed implant case series. All consecutive cases of a single implant-supported crown or a 3-unit implant-supported prosthesis (FDP/bridge) with an implant at each end and one intervening pontic placed in the anterior and/or posterior region were included. As treatment was non-randomised and the study used secondary data from one clinic in DHA, which was pooled, individual consent for the study was not required, although all patients consented to the treatment. The study was approved by the Dubai Research Ethics Committee (Ref: DSREC-SR-03/2018_04).

Data was collected from patients’ electronic records in Dubai Health Authority (DHA) using the D4W dental practice management software (Dental4Windows, Centaur Ltd, Australia). The anonymised patient demographic data included gender, age, medical history, patients’ smoking habits and number of remaining teeth excluding the third molars. To be included, all implant prostheses had to be in function for at least 1 year. Cantilevered implants, implants supporting complete or removal partial dentures and any implant site that received bone graft material or a sinus lift procedure were excluded. Patients on bisphosphonate therapy or who had bone disease were excluded but patients with any other medical condition were included. Implant information regarding implant manufacturer, position, dimensions, time of insertion, time of loading, age of the implant and type of anchorage were entered into the database. Case type was dichotomised into single implant crown or implant-supported dental prosthesis (FDP). Biological complications included bone height mesially and distally, periapical radiolucency, implant mobility, peri-implantitis, and implant removal. Prosthetic complications included screw loosening, screw fracture, ceramic chipping, de-cementation of implant crown and any re-make of the prosthesis. Implants ‘In function’ in this study were defined as an implant and prostheses in situ regardless of the presence or absence of biological or technical complications.

Crestal bone height was measured on standardised peri-apical digital radiographs using Digora software. To minimise measurement error all radiographs were taken with the long cone parallel technique with a Rinn ORA positioner (Dentsply Sirona, Charlotte, NC 28277) a source to skin distance of 200 mm (8 inches) and a Planmeca ProSensor® HD intra-oral digital sensor (Planmeca Oy, FI-00880 Helsinki, Finland) placed in a Rinn XCP DS Fit (Dentsply Sirona) universal sensor positioner whilst the patient was biting into the bite block. Mesial and distal bone height was measured from the implant abutment interface to the most apical area of bone implant contact by one examiner (SAH) who was not involved in the patient’s treatment. For the FDP cases, the mesial and the distal aspects of each implant were measured. The first radiograph was taken at the time of prosthetic placement as a baseline measurement and the second radiograph was taken at least one-year post prosthesis fitting but no more than 18 months post-placement. Ten randomly selected baseline radiographs had bone height measurements performed twice, at least two weeks apart, in order to determine the reliability of this measurement and calculate an Intra-examiner Correlation Co-efficient (ICC). Standard oral and implant hygiene instructions such as flossing and interdental brushing were given to all patients who were requested to attend for annual follow-up.

Statistical analysis was performed using SAS software (version 9.4; SAS Institute, Cary, NC, USA). The associations of outcomes with patient and implant level factors were assessed using mixed regression models, adjusting for the clustering of implants within patients. Mixed linear regression models were used for crestal bone loss, and mixed logistic regression models for complications. Univariable models were fitted for each factor, and also multivariable models including all patient and implant level factors.

## Results

The Intraclass Correlation Coefficient (ICC) was 0.84 which is considered good. A total of 454 cases (66% female and 34% male) met the inclusion criteria with 1673 implants having been placed. Table 1 shows that males had a mean age of 54.0 years compared to 49.0 years for females. The mean number of teeth present in the mouth of females was 24.0 and for males this was 23.3.

**Table 1:** Age of the participants by gender

Gender	N	Mean	Std. deviation
Age			
Females	302	49.0	12.9
Males	152	54.0	14.6

The characteristics of the included cases are shown in Table 2. Mean implant age, defined in this study as the time from implant placement until the time of data collection was 4.9 years and the mean time from implant insertion to prosthesis placement/loading was 8.8 months. The first bone height radiographic measurement was at prosthesis placement and all second bone readings were taken one year after prosthesis placement. Mean age of single implants (4.8 years, SD 2.2) was similar to implants supporting a FDP (5.0 years, SD 2.3).

**Table 2:** Characteristics of the cases included in the study

Characteristics	Frequency	Percent (%)
Medically fit	291	64
Medical condition present	163	36
Non-smoker	396	87
Smoker	58	13

Implants were grouped into two categories as either single crown cases or FDP cases but it should be noted that 111 cases had a mixture of both single and implant-supported bridges (FDPs). The implant abutment interface connection was categorised into screw or cement and four different implant manufacturers were used by the operators who placed the implants (Table 3).

**Table 3:** Implant and operator data

	Frequency	Percent (%)
Case type by implant		
Single	780	46.6
FDP	893	53.4
Implant–abutment connection		
Screw type	1607	96.1
Cement type	66	3.9
Implant manufacturer		
Astra	51	3
Xive	485	29
Ankylos	1125	67.2
Frialit	12	0.7
Operator by patient		
Maxillofacial surgeons	217	48
Restorative (periodontist and prosthodontist)	237	52

The most frequent sites for implant placement were the lower left and right posterior sextants while the least common site was the lower anterior sextant (Table 4).

**Table 4:** Position of implant in the oral cavity

Implant position	Frequency	Percent (%)
Upper right posterior sextant	347	20.7
Upper anterior sextant	176	10.5
Upper left posterior sextant	277	16.6
Lower left posterior sextant	389	23.3
Lower anterior sextant	92	5.5
Lower right posterior sextant	392	23.4
Total	1673	100

Tables 5 and 6 shows mean mesial and distal crestal bone loss according to several study variables, and results from both unadjusted and adjusted regression analyses.

**Table 5:** Mean crestal bone loss (mesial) one year after placement by study variables

Study variables (pts = number of patients^a^, n = number of implants)	Mean crestal bone loss mesially (SD) mm	Unadjusted β (95% CI)^b^ p value	Adjusted β (95% CI)^c^ p value
Gender			
Male (pts = 152, n = 610)	0.78 (0.73)	Ref	Ref
Female (pts = 302, n = 1063)	0.72 (0.66)	− 0.06 (− 0.17, 0.04)	0.00 (− 0.09, 0.09)
		p = 0.233	p = 0.963
Age			
20–40 (pts = 124, n = 332)	0.63 (0.72)	Ref	Ref
41–60 (pts = 198, n = 751)	0.68 (0.64)	0.06 (− 0.06, 0.18)	− 0.10 (− 0.19, 0.00)
> 60 (pts = 132, n = 590)	0.89 (0.70)	0.26 (0.13, 0.39)	− 0.04 (− 0.15, 0.07)
		p < 0.001	p = 0.104
Case type			
Single crown (n = 780)	0.30 (0.43)	Ref	Ref
FDP group (n = 893)	1.13 (0.63)	0.86 (0.80, 0.92)	0.86 (0.81, 0.92)
		p < 0.001	p < 0.001
Medical condition			
No (pts = 291, n = 1031)	0.67 (0.66)	Ref	Ref
Yes (pts = 163, n = 642)	0.86 (0.71)	0.17 (0.07, 0.27)	0.07 (− 0.01, 0.15)
		p = 0.001	p = 0.080
Smoking			
No (pts = 396, n = 1482)	0.75 (0.67)	Ref	Ref
Yes (pts = 58, n = 191)	0.68 (0.79)	− 0.01 (− 0.16, 0.14)	0.00 (− 0.13, 0.12)
		p = 0.905	p = 0.988
Implant abutment connection			
Screw (n = 1607)	0.75 (0.69)	Ref	Ref
Cement (n = 66)	0.56 (0.56)	− 0.14 (− 0.33, 0.04)	− 0.06 (− 0.21, 0.08)
		p = 0.128	p = 0.394
Implant position (sextant)			
UR posterior (n = 347)	0.77 (0.73)	Ref	Ref
Upper anterior (n = 176)	0.70 (0.60)	− 0.10 (− 0.21, 0.02)	− 0.15 (− 0.25, − 0.05)
UL posterior (n = 277)	0.73 (0.69)	− 0.01 (− 0.11, 0.09)	0.03 (− 0.05, 0.11)
LL posterior (n = 389)	0.72(0.70)	− 0.08 (− 0.17, 0.01)	− 0.01 (− 0.09, 0.06)
Lower anterior (n = 92)	1.16 (0.68)	0.28 (0.13, 0.42)	0.12 (0.00, 0.25)
LR posterior (n = 392)	0.67 (0.64)	− 0.08 (− 0.17, 0.01)	− 0.01 (− 0.09, 0.06)
		p < 0.001	p = 0.002
Implant manufacturer			
Ankylos (n = 1125)	0.75 (0.69)	Ref	Ref
Astra (n = 51)	0.46 (0.50)	− 0.30 (− 0.51, − 0.08)	− 0.30 (− 0.47, − 0.13)
Xive (n = 485)	0.76 (0.69)	0.07 (− 0.03, 0.17)	0.04 (− 0.04, 0.12)
Frialit (n = 12)	0.58 (0.63)	− 0.26 (− 0.67, 0.15)	− 0.23 (− 0.56, 0.09)
		p = 0.008	p < 0.001
Operator			
Maxillofacial (pts = 217, n = 854)	0.78 (0.70)	Ref	Ref
Restorative (pts = 237, n = 819)	0.70 (0.68)	− 0.02 (− 0.12, 0.08)	0.09 (0.02, 0.17)
		p = 0.666	p = 0.018

^a^Number of patients (pts) is only presented for patient level variables

^b^Estimates and confidence intervals derived from mixed linear regression models, implants clustered within patients

^c^Estimates and confidence intervals derived from mixed linear regression models, implants clustered within patients, adjusted for all other factors in the table

**Table 6:** Mean crestal bone loss (distal) one year after placement by study variables

Study variables (pts = number of patients, n = number of implants)^a^	Mean crestal bone loss distally (SD) mm	Unadjusted β (95% CI) p value^b^	Adjusted β (95% CI) p value^c^
Gender			
Male (pts = 152, n = 610)	0.93 (0.85)	Ref	Ref
Female (pts = 302, n = 1063)	0.82 (0.75)	− 0.11 (− 0.23, − 0.00)	− 0.08 (− 0.18, 0.01)
		p = 0.048	p = 0.094
Age			
20–40 (pts = 124, n = 332)	0.74 (0.85)	Ref	Ref
41–60 (pts = 198, n = 751)	0.80 (0.77)	0.08 (− 0.06, 0.21)	− 0.10 (− 0.20, 0.01)
> 60 (pts = 132, n = 590)	0.99 (0.75)	0.26 (0.18, 0.40)	− 0.09 (− 0.21, 0.03)
		p < 0.001	p = 0.168
Case type			
Single crown (n = 780)	0.36 (0.54)	Ref	Ref
FDP group (n = 893)	1.29 (0.71)	0.93 (0.87, 1.00)	0.94 (0.87, 1.01)
		p < 0.001	p < 0.001
Medical condition			
No (pts = 291, n = 1031)	0.78 (0.77)	Ref	Ref
Yes (pts = 163, n = 642)	0.98 (0.80)	0.18 (0.07, 0.29)	0.08 (− 0.01, 0.17)
		p = 0.002	p = 0.071
Smoking			
No (pts = 396, n = 1482)	0.87 (0.78)	Ref	Ref
Yes (pts = 58, n = 191)	0.78 (0.87)	− 0.03 (− 0.19, 0.14)	− 0.08 (− 0.22, 0.06)
		p = 0.728	p = 0.290
Implant abutment connection			
Screw (n = 1607)	0.86 (0.80)	Ref	Ref
Cement (n = 66)	0.77 (0.56)	− 0.03 (− 0.24, 0.18)	0.05 (− 0.23, 0.12)
		p = 0.761	p = 0.542
Implant position (sextant)			
UR posterior (n = 347)	0.91 (0.84)	Ref	Ref
Upper anterior (n = 176)	0.87 (0.67)	− 0.08 (− 0.22, 0.05)	− 0.16 (− 0.28, − 0.05)
UL posterior (n = 277)	0.83 (0.78)	− 0.07 (− 0.19, 0.04)	− 0.03 (− 0.12, 0.07)
LL posterior (n = 389)	0.82 (0.83)	− 0.06 (− 0.17, 0.05)	− 0.06 (− 0.15, 0.03)
Lower anterior (n = 92)	1.25 (0.80)	0.22 (0.04, 0.39)	0.05 (− 0.10, 0.20)
LR posterior (n = 392)	0.76 (0.73)	− 0.14 (− 0.24, 0.03)	− 0.06 (− 0.15, 0.03)
		p = 0.002	p = 0.065
Implant manufacturer			
Ankylos (n = 1125)	0.67 (0.51)	Ref	Ref
Astra (n = 51)	0.88 (0.80)	− 0.15 (− 0.40, 0.09)	− 0.16 (− 0.36, 0.04)
Xive (n = 485)	0.86 (0.79)	0.08 (− 0.04, 0.19)	0.06 (− 0.03, 0.15)
Frialit (n = 12)	0.83 (0.83)	− 0.10 (− 0.57, 0.38)	− 0.06 (− 0.44, 0.33)
		p = 0.256	p = 0.159
Operator			
Maxillofacial (pts = 217, n = 854)	0.89 (0.78)	Ref	Ref
Restorative (pts = 237, n = 819)	0.82 (0.80)	− 0.02 (− 0.09, 0.13)	0.11 (0.02, 0.19)
		p = 0.729	p = 0.017

^a^Number of patients (pts) is only presented for patient level variables

^b^Estimates and confidence intervals derived from mixed linear regression models, implants clustered within patients

^c^Estimates and confidence intervals derived from mixed linear regression models, implants clustered within patients, adjusted for all other factors in the table

Bone height was measured on radiographs at baseline when the prosthesis was placed and 1-year after prosthesis placement. Adjusted analyses for bone loss by gender found no differences although in the unadjusted analysis, males had significantly greater mean crestal bone loss distally (p = 0.048) but not mesially compared to females. Patient’s age was divided into three groups as follows: young (20–40 years), middle (41–60 years), and older (> 60 years). Mean crestal bone loss mesially and distally increased with age but this was not significant in the adjusted analysis (Tables 5, 6). Mean crestal bone loss in the FDP group was 1.13 mm mesially and 1.29 mm distally which was significantly greater than in the single implant-supported crown group.

After adjusting for all factors, mean mesial and distal crestal bone loss in patients with a medical condition was not significantly greater compared to medically fit patients (Tables 5, 6). There was no significant difference in mean mesial or distal crestal bone loss between smokers and non-smokers. (Table 5).

Implants placed in the lower anterior sextant, had significantly greater crestal bone loss mesially and distally compared to the other sextants in the unadjusted analysis, although the adjusted value for distal bone loss was not significant (p = 0.065) (Tables 5, 6). Mean mesial crestal bone loss around Ankylos and Xive implants was greater than around Astra and Frialit but there was no difference distally. Cases treated by maxillofacial surgeons exhibited significantly greater crestal bone loss mesially and distally compared to restorative operators after adjusted analysis for all variables. Although there was little difference between maxillofacial surgeons and restorative operators in the unadjusted analysis, after adjusting for all other factors there was a statistically significantly higher bone loss in patients treated by restorative operators.

Of the 1673 implants, 83% had no complication whereas only 17% had a complication. Furthermore, 96.1% of implants were still in function and 3.1% required remake of the prosthesis. Only 0.8% had to be removed as shown in Table 7.

**Table 7:** Distribution of implant complications and final outcomes

	Frequency	Percent (%)
No complications	1389	83
Complications		
Implant mobility	6	0.4
Peri-implantitis	9	0.5
Screw loosening	193	11.5
Screw fracture	12	0.7
Chipped ceramic	19	1.1
Crown de-cementation	45	2.7
Final outcome		
In function	1608	96.1
Remake prostheses	52	3.1
Removal of implant	13	0.8

Table 8 shows that significantly more complications occurred in the FDP group (n = 177) compared to the implant-supported crowns (n = 107). Patients with common medical conditions such as diabetes, hypertension and cardio-vascular disease did not have significantly more implant complications compared to fit and healthy patients (Table 8). Implant manufacturer and operator had no significant difference on implant complications in both adjusted and unadjusted analyses whilst implant position was only significant in the unadjusted analysis. Cement retained prostheses had significantly more than expected implant complications compared to screw retained prostheses (Table 8).

**Table 8:** Frequency of implant complications by study variables

Study variables	No complication	Complication	Unadjusted odds ratio (95% CI)	Adjusted odds ratio (95% CI)*
Gender				
Male	489 (80%)	121 (20%)	Ref	Ref
Female	900 (85%)	163 (15%)	0.83 (0.52, 1.31)	0.65 (0.36, 1.18)
			p = 0.411	p = 0.159
Age				
20–40	277 (83%)	55 (17%)	Ref	Ref
41–60	620 (83%)	131 (17%)	1.10 (0.63, 1.94)	0.93 (0.50, 1.73)
> 60	492 (83%)	98 (17%)	0.96 (0.52, 1.76)	0.71 (0.34, 1.48)
			p = 0.859	P = 0.616
Case type				
Single crown	673 (86%)	107 (14%)	Ref	Ref
FDP group	716 (80%)	177 (20%)	1.48 (1.03, 2.14)	1.74 (1.16, 2.61)
			p = 0.036	p = 0.008
Medical condition				
No	873 (85%)	158 (15%)	Ref	Ref
Yes	516 (80%)	126 (20%)	1.49 (0.95, 2.33)	1.64 (0.96, 2.79)
			p = 0.086	p = 0.071
Smoking				
No	1222 (82%)	260 (18%)	Ref	Ref
Yes	167 (87%)	24 (13%)	0.68 (0.34, 1.39)	0.58 (0.25, 1.38)
			p = 0.293	p = 0.220
Implant abutment connection				
Screw	1369 (85%)	238 (15%)	Ref	Ref
Cement	20 (30%)	46 (70%)	29.32 (12.56, 68.41)	36.07 (14.59, 89.18)
			p < 0.001	p < 0.001
Implant position (sextant)				
UR posterior	292 (84%)	55 (16%)	Ref	Ref
Upper anterior	134 (76%)	42 (24%)	1.90 (1.04, 3.48)	1.14 (0.59, 2.23)
UL posterior	232 (84%)	45 (16%)	0.87 (0.52, 1.48)	0.79 (0.45, 1.37)
LL posterior	328 (84%)	61 (16%	1.16 (0.70, 1.91)	1.14 (0.68, 1.93)
Lower anterior	83 (90%)	9 (10%)	0.59 (0.23, 1.48)	0.34 (0.12, 1.00)
LR posterior	320 (82%)	72 (18%)	1.49 (0.92, 2.42)	1.40 (0.84, 2.35)
			p = 0.040	p = 0.084
Implant manufacturer				
Ankylos	43 (84%)	8 (16%)	Ref	Ref
Astra	418 (86%)	67 (14%)	0.86 (0.31, 2.44)	0.84 (0.26, 2.70)
Xive	917 (82%)	208 (18%)	0.85 (0.53, 1.37)	0.80 (0.46, 1.38)
Frialit	11 (92%)	1 (8%)	0.60 (0.05, 7.04)	0.23 (0.01, 5.21)
			p = 0.893	p = 0.695
Operator				
Maxillofacial	703 (82%)	151 (18%)	Ref	Ref
Restorative	686 (84%)	133 (16%)	1.03 (0.67, 1.61)	1.39 (0.82, 2.35)
			p = 0.883	p = 0.216

^*^Estimates and confidence intervals derived from mixed logistic regression models, adjusted for clustering of implants within patients

## Discussion

The Emirate of Dubai had a total population of 2,976,455 in 2017, of which 74.9% were males in the working age category of 20–50 years [20]. It is interesting to note that females accounted for 60% of the patients who received implant treatment in the dental centre in Dubai, despite the fact that all Emirati nationals are entitled to receive free implant treatment. Females are more proactive regarding oral health compared to males which would account for the higher number of females in the present study.

In the present study, biological complications were categorized as mesial and distal crestal bone loss one year after prosthesis placement, periapical radiolucency, implant mobility and implant removal. Prosthetic complications were screw loosening, screw fracture, ceramic chipping, de-cementation and re-make of the implant prosthesis.

Factors that could affect marginal bone included gender, age, medical condition, smoking, operator, implant manufacturer, implant anchorage, implant position and case type. Previously reported bone loss after 1 year in function averaged between 1.5 and 2 mm and thereafter annual bone loss was 0.2 mm [21]. In the present study, after a year in function, mean marginal bone loss around single implant supported crowns was 0.30 mm mesially and distally 0.36 mm, whereas in the 3-unit implant supported prostheses (FDPs), mean marginal bone loss was 1.13 mm mesially and 1.29 mm distally. While both types of implant restoration had bone loss within the range reported by Albrektsson et al. [21], the implant supported prostheses had significantly more bone loss after one year. Although outcome measures differ, this conflicts with the results of Ravida et al. who found that patients with a 3-unit implant supported bridge had a 72% lower risk of developing peri-implantitis compared to 3 non-splinted crowns next to each other and that survival of 3-unit implant supported bridges (FDPs) was better than both 3 adjacent single (non-splinted) implant-supported crowns and 3 splinted implant-supported crowns over the mean 76-month follow-up period [5].

In the systematic review conducted by Pol and co-workers, the annual failure rate of 3-unit tooth supported FDPs was higher at 3.6% than 3-unit implant supported FDPs (2.6%) [4]. In this study, the greater bone loss at one year in 3-unit implant supported FDPs compared to single crowns is intuitive in so far as plaque control is more difficult under bridge pontics. To better understand this relationship, the bone height on the side of the implant adjacent to the pontic, which is more difficult to clean, should be assessed and compared to the side away from the pontic.

The results presented here found that implant-supported fixed prostheses have significantly greater mesial and distal marginal bone loss compared to single implant-supported prostheses. The reasons for this are not fully understood but inter-proximal hygiene is more difficult around implant-supported fixed prostheses [22, 23]. Although survival data is not presented here, our results are intuitive and indicate single implant-supported crowns are likely to have better survival, at least in the short term, compared to implant-supported FDPs because less bone is lost.

Although males had lost more bone distally compared to females, this was not significant in the adjusted analysis. Poor access for oral hygiene on the distal aspect of the implant, greater forces distally and greater bite force in males than in females [24, 25] may account for this. Also, the males in this study were significantly older than the females and it is well known that age influences crestal bone loss and, as visual acuity and manual dexterity decline, maintaining oral health becomes more difficult.

The medical conditions were gained from the hospital records and 163 patients in this study had either diabetes, hypertension and/or cardiovascular disease. None of the patients had neoplastic or metabolic bone disease. Contraindications to the placement of implants in medically compromised patients are relative rather than absolute. Implants are a feasible treatment option in almost any medically compromised patient, but excellent plaque control, regular maintenance and follow-up are required [26]. Even in patients with altered bone metabolism such as osteoporotic individuals, implant survival is comparable to those seen in healthy individuals [27, 28]. The bidirectional relationship between diabetes and periodontal disease can increase the risk of peri-implantitis and impair osseointegration in poorly controlled cases but when diabetes is well controlled, implant survival is similar to healthy patients within the first 6 years, but survival reduces over 20 years [29]. In the present study after adjustment for all variables, medical conditions did not result in significantly greater crestal bone loss compared to healthy individuals. This supports implant provision in medically compromised patients. Diz and colleagues reviewed a range of medical problems and concluded that the degree of disease control may be far more important than the nature of the systemic disorder itself [30]. This confirms the previous finding that age, sex, ASA classification and immediate implants were not predictive for bone loss within the first year after implant placement [31]. The medication profile of this group of patients was not investigated but future research should study the association between medicines and implant outcome as medication such as glucocorticoids, non-steroidal anti-inflammatories and chemotherapy can adversely affect wound healing [32].

Significant bone loss has been reported in heavy smokers (> 15 cigarettes per day) after 4 years [33]. Conversely, Calvo-Guirado et al. [34] reported that smoking had no negative influence on crestal bone loss after a 5 year follow up. The effect of smoking on implant survival is still controversial but it is not a contraindication for implant therapy. The prevalence of smoking in the Middle East has increased, especially in young adults with the added influence of poly-tobacco use including midwakh/dokha and water pipes (shisha) [35–37]. In the present study, there was no significant difference on crestal bone between smokers and non-smokers. This can be explained by the low number of smokers although under-reporting for cultural reasons, especially among females has been recognised [37].

Despite the adjusted analysis finding that marginal bone loss was significantly greater in cases treated by restorative operators compared to maxillofacial surgeons, the difference of 0.1 mm, is not clinically significant (Tables 5, 6). Maxillofacial surgeons were assigned to more complex cases which could account for a greater risk of problems although it has been shown that more experienced surgeons have higher survival rates [38].

Lemos et al. and Hameed et al. reported that screw retained prostheses had greater crestal bone loss than cement retained, which was found in the present study but the difference was not significant probably because of the low number of cement retained implants at just 66 [39, 40].

Furthermore, the present study agrees with Ajanović et al. [41], who reported that the anterior mandible has the highest crestal bone loss. We report a mean 1.2 mm of bone loss in the lower anterior region after 1 year of function, which was significantly greater than the other sites mesially but not distally when adjusted for all variables. The anterior part of the mandible is narrow and mainly composed of cortical bone and has less cancellous bone and requires more force to drill, leading to excessive heat generation and crestal bone loss [42].

In the present study, 96.1% of the implants were still in function with only 3.1% requiring remake and 0.8% needed removal. Thus, both the single implant-supported prostheses and the implant-supported fixed prostheses had good survival. This agrees with Pjetursson et al. [43] who estimated the survival rate of implants supporting fixed prostheses was 95.6% after 5 years and 93.1% after 10 years. The estimated survival rate after 5 years function in single implant supported crowns was 94.5% [44], while others estimated the survival rate after 10 years of function in fixed–fixed implant supported prostheses was 86.7% [1], which is lower than that reported in the present study.

Screw loosening was the most frequent complication (11.5%) in this study followed by crown de-cementation (2.7%). Pjetursson et al. [43] reported that veneering material fracture was the most frequent complication (13.5%) followed by 5.3% screw loosening. Because physical properties of veneering ceramic have evolved and improved, chipping is less of a problem nowadays.

The association between complications and gender, age, medical condition and habits is less understood. In this study, implant complications were not significantly different by gender, age, medical condition, smoking, implant manufacturer and operator. Even though there were only 66 cemented cases, implant complications were higher than expected in cement retained prostheses (70%) compared to screw retained prostheses (15%), which is similar to previous findings in so far cement retained prostheses had more biological complications, whereas screw retained had more technical complications [45]. Screw retained prostheses require meticulous surgical technique and prosthetic design whilst cement retained prostheses provide optimal occlusal design, superior aesthetics and passive fit of the restoration [46–48].

A recent systematic review found no statistically significant difference in prosthetic complications among single implant-supported prostheses and implant-supported fixed prostheses [48]. Others have shown that most prosthetic complications among implant-supported fixed prostheses were chipped ceramic [9, 47]. Most prosthetic complications among single implant-supported prostheses were screw loosening, ceramic chipping and loss of retention [9, 47] whilst most technical complications in implant-supported fixed prostheses are fractures of the veneering material, abutment or screw loosening, and loss of retention [44]. The present study found 20% of implant-supported fixed prostheses had complications which was significantly greater than the 14% of single implant-supported prostheses. Only 1% had chipped ceramic as monolithic Zirconia was used in most cases. Furthermore, the most common prosthetic complication in the present study in both single implant-supported prostheses and implant-supported fixed prostheses was screw loosening which is in line with previous studies. Increased screw loosening has been associated with increased inter-arch distance, increased ratio of crown-to-implant length, inadequate tightening torque and stress generation that changes the screw geometry, causing metal fatigue and ultimately leading to screw loosening [49, 50].

The main limitation of the present study is its retrospective design and reliance on the accuracy of patient records. Furthermore, marginal crestal bone loss around implants occurs in a three-dimensional manner whilst the radiographic assessment only allows measurement of the mesial and distal surface but not the buccal and lingual.

## Conclusion

This large-scale study of 1673 implants in 454 subjects found that 3-unit implant-supported prostheses (FDPs) with one intervening pontic had significantly more marginal crestal bone loss compared to single crown supported implants one year after loading. This implies that clinicians should be vigilant and careful periodontal/bone assessment around implant supported FDPs is needed. Implant position and operator were associated with bone loss, but gender, patient’s age and medical condition were not. Fixed dental prostheses and their supporting implants had significantly more complications compared to single crowns on implants. Cemented implants had more complications than screw retained.

## Data Availability

The data that support the findings of this study are available from Dubai Health Authority but restrictions apply to the availability of these data, which were used under license for the current study, and so are not publicly available. Data are however available from the authors upon reasonable request and with permission of Dubai Health Authority.

## Abbreviations

DHA: Dubai Health Authority
D4W: Dental4Windows, Centaur Ltd, Australia
